# Everybody's Page

**Published:** 1889-02-23

**Authors:** 


### Everybody's Page.

Dr. Desjardins, a French doctor, resident in Constantinople, has established a hydropathic establishment, "Cadikew," in Turkey.

An American doctor, Dr. Cryer, says that he has among his patients members of the same family, representing five generations, all of whom are congenitally wanting in one of the lower incisor teeth.

In the South of France they have a proverb to the effect that "where the sun does not enter the doctor does." Fresh air is a good thing, but fresh air combined with sunlight is a better.

Mr. T. Assheton Smith, a Calcutta veterinary surgeon, has applied the principle of skin-grafting to horses. He tried the experiment on a pony which had skinned its leg from knee to fetlock. As the wound got older the graft succeeded better, and the skins which Mr. Smith found most efficacious were those of frogs and chickens.

A recent order of the Minister of the Interior of the Ottoman Empire commands that the diploma of every doctor must be returned on his death to the college which granted it. This decree has been made necessary by the fact that many quacks bought the diplomas of deceased physicians, and practised on the strength of possessing them.

There are female physicians in Turkestan, but there is so little demand for their services that they have to pay patients to employ them. A similar prejudice against female doctors exists in some parts of Siberia, and every woman who enters the hospital at Orusk receives a present of three roubles. This dislike does not seem to be shared by the inhabitants of Russia in Europe.

Charles Lever, the incomparable Irish novelist, who was a medical man--not a soldier, as one would imagine the author of "Harry Lorrequer" must have been—was a firm believer in bleeding. In any ailment of his own at least, he turned for help to leeches, if not to the lancet. Once when his publisher's neglect to answer his letters had upset him, he said it would cost him a pint of colchicum and a rivulet full of enraged leeches.

In the dry air of Buenos Ayres the dead bodies of animals do not decompose, but simply dry up. This simple method of desiccation was practised in Peru, where the bodies of the Incas were thus preserved to perfection, without so much as a hair or an eyebrow being wanting. "In the great temple of the sun at Cuzco," says Garcilaso, "their bodies, ranged on one side, and those of their queens on the other, sat, clothed in their former princely attire, upon chairs of gold their heads inclined downwards, covered with raven black or silver white hair, and their hands placidly crossed on their bosoms."

In the early eays of emigration very little attention was paid to the health and comfort of the passengers, and the death-rate on board the ships was enormous. Going from England to New South Wales 100 passengers out of 306 died on board the *Hillsborough*, while on one voyage of the *Atlas* there were 61 deaths among 175 passengers. The first step towards a remedy for this terrible state of things came from self-interest. A new form of contract was introduced, by which the emigrant did not pay for his passage on embarking but on arriving at his journey's end; consequently the companies received payment only for those who were landed alive at the end of the voyage. From that time emigrant ships began to carry surgeons, and the hygienic condition of the emigrants and their surroundings was carefully attended to.

There's nothing like leather, even when it has fallen so low as not to be even on its last legs. Old boots and shoes, when ground down, make good manure, being rich in nitrogen. Is anything ever quite useless? Does not this last discovery seem to predict the reign of an agricultural Buddha, with endlessly protean possibilities of incarnation? Doubtless, in time, when the folk-lore robbers have stolen all our nursery stories from us to turn them into dreary nature-myths—when they have done their wicked worst with Cinderella and Bluebeard, they will take up the legend of the famous cook who "could make ragoûts, even of old shoes," and make that into a myth about an ingenious female agriculturist—of the patriarchal, or matriarchal, period beloved by Mrs. Mona Caird—who ground down the cast-off boots of her household for manure, raised crops on the soil so enriched, and made the grain so produced into bread and other food for her family.

"Miranda," in the *Lady's Pictorial*, has a finely satiric note on the "abbreviated Latin and snakes' tails" of doctors' prescriptions, with special reference to the recent accident at University College Hospital. "Can an old custom be given up because it occasionally entails the sacrifice of a human life? Certainly not," she exclaims, and indulges in the venerable, the decrepit, and toothless joke that if people only knew the contents of their medicine bottles, they would not swallow them nor think them of any value. Perhaps they wouldn't; Carlyle's thirty millions have not yet died off. But the doctors are by no means so much set on secresy as "Miranda" thinks. If the average Board school pupil does not comprehend that "aquam ad ℥vi." does not mean "water to the amount of six ounces," that pupil shamefully neglects his opportunities. But it is convenient for doctors and chemists all the world over to have a common tongue for intercourse, as perhaps "Miranda" would believe if she wanted a prescription given her by an English doctor made up in Paris or St. Petersburg. But we don't want to be too hard on "Miranda." Her weekly notes in the *Lady's Pictorial* shows her to be a constant reader of our pages, and one who profits by the information she finds there.

Dr. Paul Moreau, in a work on insanity in children, has given the name of "psychical epidemics" to these occasional outbursts of emotional activity which, in one form or another, affect children under the influence of some excitable master mind. The most notable instance of such an infectious enthusiasm was the children's crusade. In 1212 a young shepherd named Etienne, a native of the village of Cloyes, resolved, under the influence of the ideas then popular, to set out to recover the Holy Sepulchre. He called his companions to go with him, and the infection of religious excitement spread throughout both France and Germany, where a boy of twelve, named Nicholas, led the band. Children of ten or twelve years of age, both boys and girls, joined the band, indifferent alike to home duties and home affections. An army of 50,000 children set out for the Holy Land; but they had neither money nor provisions; they lived on wild fruits and roots, and they were in a state of intense religious excitement. The result was hunger, fatigue, hysteria, convulsions, death. Thousands fell by the way, yet the remainder persevered in their attempt. They collected at Marseilles, expecting that the sea would dry up and afford them a passage to Palestine. They waited in vain for the miracle, and some of the weaker and more sceptical set out for their deserted homes, while others wandered into the neighbouring districts. A faithful remnant kept true to their purpose, and gladly accepted the offer of two merchants to give them a passage to the Holy Land. They were taken there according to promise, but only to be sold as slaves to the Saracens. Such was the pitiful end of this "psychical epidemic."

